# Esophageal adenocarcinoma models: a closer look

**DOI:** 10.3389/fmolb.2024.1440670

**Published:** 2024-11-12

**Authors:** Nadeem Bhat, Marwah Al-Mathkour, Selma Maacha, Heng Lu, Wael El-Rifai, Farah Ballout

**Affiliations:** ^1^ Department of Surgery, Miller School of Medicine, University of Miami, Miami, FL, United States; ^2^ Department of Veterans Affairs, Miami Healthcare System, Miami, FL, United States; ^3^ Sylvester Comprehensive Cancer Center, Miller School of Medicine, University of Miami, Miami, FL, United States

**Keywords:** animal models, patient-derived organoids, esophageal adenocarcinoma, GEMMs, 3D culture models

## Abstract

Esophageal adenocarcinoma (EAC) is a subtype of esophageal cancer with significant morbidity and mortality rates worldwide. Despite advancements in tumor models, the underlying cellular and molecular mechanisms driving EAC pathogenesis are still poorly understood. Therefore, gaining insights into these mechanisms is crucial for improving patient outcomes. Researchers have developed various models to better understand EAC and evaluate clinical management strategies. However, no single model fully recapitulates the complexity of EAC. Emerging technologies, such as patient-derived organoids and immune-competent mouse models, hold promise for personalized EAC research and drug development. In this review, we shed light on the various models for studying EAC and discuss their advantages and limitations.

## Graphical Abstract

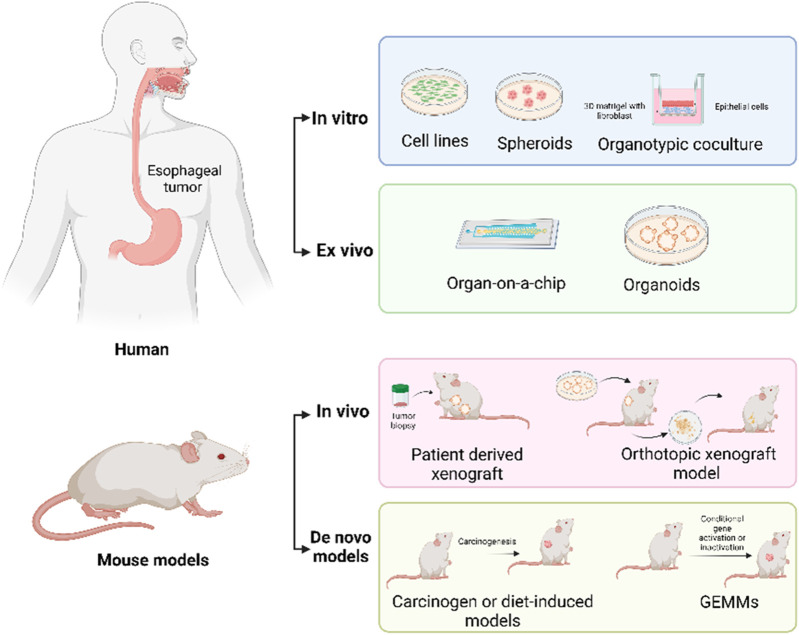

## 1 Introduction

Esophageal cancer (EC) is the tenth most common cancer worldwide and the sixth leading cause of cancer deaths (Sung et al., 2021). It is characterized by its high mortality rate and poor prognosis at the time of diagnosis. Although esophageal squamous cell carcinoma (ESCC) is the most prevalent histological type worldwide, EAC has one of the fastest-growing incidences among cancers in the United States and industrialized world. EAC is quickly becoming the most prevalent type of EC in Western countries (Uhlenhopp et al., 2020). Moreover, EAC has one of the lowest survival rates due to therapeutic resistance and limited effective treatment options (Sung et al., 2021).

The risk factors for developing EAC include Barrett’s esophagus (BE), gastroesophageal reflux disease, obesity, and tobacco consumption. BE develops in response to an inflammatory microenvironment caused by chronic reflux and is characterized by the pathological replacement of esophageal squamous epithelium by columnar epithelium (Shaheen et al., 2022). BE is widely considered a precancerous lesion of EAC that can progress to low-grade dysplasia, high-grade dysplasia, and finally to EAC. Similar to EAC, the incidence of BE has been increasing in the Western countries (Coleman et al., 2011).

Cancers originating in the context of chronic inflammation such as EAC are likely driven by environmental factors and stromal cell interactions, which together form an aberrant pro-inflammatory tumor microenvironment (TME) that predisposes to cancer initiation and tumor progression (Lin et al., 2016). The TME of EAC is complex composed of immune cells, fibroblasts, and the extracellular matrix that supports all steps of tumorigenesis (Lin et al., 2016). Therefore, to design better therapeutic strategies for EAC, it will be of paramount importance to understand the role and contribution of the tumor microenvironment.

Despite major advances in targeted treatments in other cancers, the progress in EAC has been limited to VEGF and HER2 inhibitors in clinical practice (Yang et al., 2020; Wilke et al., 2014; Fuchs et al., 2014; Safran et al., 2022). More recently, immune checkpoint inhibitors have been tested, but durable responses are rare (Baxter et al., 2021). Overall, patients with EAC have poor clinical outcomes with dismal survival rates, requiring the development of different therapeutic options.

The tumoral heterogeneity of EAC and limited cellular and *in vivo* models that reflect the primary disease are major hurdles on the way of developing and testing novel treatments. Indeed, the lack of comprehensive EAC models that incorporate the disease’s etiology, the complexity of the TME, and the tumor’s genetic heterogeneity is a rate-limiting step in advancing EAC treatment. Improving the ability to recapitulate these aspects will certainly aid in understanding the oncogenic signaling driving EAC, testing the efficacy of new therapies, and discovering new biomarkers, therefore improving the diagnosis and clinical outcome. This review summarizes the various methods used in EAC modeling with an emphasis on their application, advantages, and limitations.

## 2 *In vitro* EAC models

**FIGURE 1:**
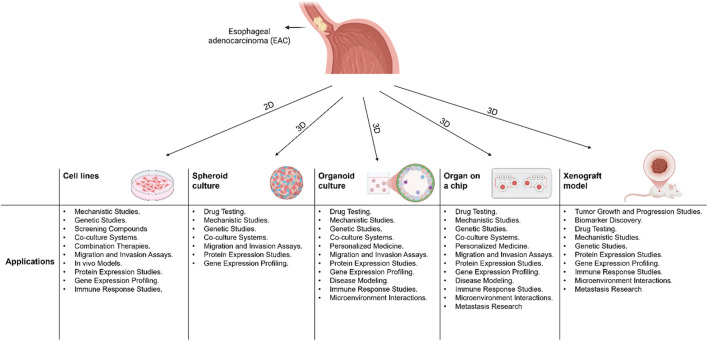
A schematic diagram showing 2D and 3D models that are used in Esophageal Adenocarcinoma (EAC) and their applications.

*In vitro* models serve as valuable tools for researchers to study cellular phenotypes, test drug effects, and explore disease mechanisms without relying heavily on animal experimentation. In the following section, we will provide an overview of the different types and applications of *in vitro* EAC models (Figure 1).

### 2.1 Cell lines

Cell lines serve as indispensable tools in EAC research, facilitating the detailed investigation of specific molecular pathways governing EAC tumorigenesis, metastasis, and response to anti-cancer therapies. Their utility extends to precise modeling of EAC progression and treatment outcomes, owing to their ease of manipulation and compatibility with both *in vitro* experiments and animal xenograft models (Boonstra et al., 2010; Katt et al., 2016). While cell lines facilitate quantitative analysis, they fall short in qualitative assessments compared to other models for several reasons including: Genetic and Phenotypic variability due to their immortalization and continuous passaging, which can lead to significant variability that does not accurately reflect the original tumor’s characteristics, making it difficult to draw reliable conclusions (Freedman et al., 2015). The lack of tumor microenvironment representation, which includes interactions with stromal cells, immune cells, and extracellular matrix components resulting in incomplete understanding of tumor behavior and drug responses (Daniela et al., 2013; Corrò et al., 2020). Reproducibility issues due to high genetic variability studies using cell lines may produce results that are not consistent when replicated in other models or clinical settings (Freedman et al., 2015). These limitations significantly impact EAC research in several ways including: 1) Lack of Biological and Physiological Relevance: traditional cell lines often fail to accurately mimic the complex biology and physiology of EAC tumors. This can lead to discrepancies between *in vitro* findings and clinical outcomes (Milne et al., 2024). 2) Tumor Heterogeneity: EAC tumors are highly heterogeneous, consisting of a diverse population of cells with different genetic and phenotypic characteristics. Traditional cell lines often represent only a small subset of this diversity, which can result in incomplete or misleading conclusions (Clemons et al., 2014). 3) Limited Availability of Representative Models: EAC-specific cell lines are relatively limited, which hampers the ability to conduct comprehensive research. The development of new cell lines, such as the OANC1, is crucial but still insufficient to cover the full spectrum of EAC biology (Milne et al., 2024). These limitations underscore the need for more advanced and representative models to improve our understanding of EAC and develop effective treatments.

### 2.2 Three-dimensional (3D) cell culture models

Three-dimensional (3D) cell culture model systems have been utilized as experimental platforms that closely mimic physiological conditions to examine esophageal biology in both normal and pathological contexts (Whelan et al., 2018). Numerous 3D culture techniques have been developed over the history of cell culture, along with special scaffolds, matrices, and media. These techniques have given scientists unique platforms to examine a variety of biological processes in the esophagus, including proliferation, differentiation, motility, stress response, and both homotypic and heterotypic cell–cell interactions of epithelial cells (Whelan et al., 2018). A range of cell types, including fibroblasts, endothelial cells, and inflammatory cells, are involved in cellular interactions in the esophageal tissue microenvironment under both homeostatic and pathologic conditions, such as an inflammatory milieu. These interactions are mediated by extracellular matrix proteins, such as matrix metalloproteinases, and cell surface molecules, such as integrins and receptors (Whelan et al., 2018). Our understanding of the molecular mechanisms and signaling pathways underlying esophageal physiology and disease has been significantly improved through experimental modifications of 3D cultures (Whelan et al., 2018). Among these 3D platforms are organ culture, organotypic tissue culture (OTC), sphere formation, organ-on-a-chip, and 3D organoid system.

#### 2.2.1 Organ culture

Organ (explant) culture was the predominant method for conducting *in vitro* analyses of live esophageal tissue until the late 1970s and early 1980s, when primary esophageal epithelial cell culture (Katayama et al., 1984) and esophageal cancer cell lines (Nishihira et al., 1993) became available. Esophageal explants from humans and animals remain vital for 3–14 days *ex vivo*, according to preliminary organ culture studies, and have provided important insights into the physiological functioning of the esophagus (Stenn and Stenn, 1976). The complexity and biology of natural tissues can be replicated with this methodology; however, sample viability is limited, and the results obtained from these methodologies can be difficult to interpret. As a result, several techniques have been developed to overcome the limitations posed by utilizing recently harvested tumor samples, while still permitting three-dimensional experiments (Lv et al., 2017; Meijer et al., 2017).

#### 2.2.2 Organotypic co-culture

Organotypic co-culture (OTC) has been primarily utilized in tissue engineering research with an emphasis on epithelial cell characteristics. This includes studies of interactions between epithelial and stromal cells after these cells have undergone genetic and pharmacological changes (Kalabis et al., 2012). Organotypic cultures are designed to mimic the complex interactions and architecture of tissues or organs. The types of cells used in these cultures can vary depending on the specific organ or tissue being modeled. Some common types of cells used in OTC include stem cells such as embryonic stem cells (ESCs), induced pluripotent stem cells (iPSCs), and adult stem cells (ASCs), primary cells directly taken from living tissues, immortalized cell lines, and tumor cells (Suarez-Martinez et al., 2022). The development of OTC involves first casting an acellular collagen matrix onto the bottom of the insert, and then casting a layer of esophageal fibroblasts combined with Matrigel and collagen type I. These two layers are cultured for up to 7 days initially, replacing the esophagus “mesenchyme” and permitting fibroblast-mediated constriction of the collagen matrix. On day five, the surface of the restricted matrix is seeded with epithelial cells. Every 2 days, the OTC medium is replaced, and the epithelium is exposed to air to form a liquid-air interface that promotes epithelial stratification and differentiation. Finally, on day 15, the resultant OTC may be used for histological processing, followed by immunohistochemistry or immunofluorescence (Kalabis et al., 2012). It is also possible to peel the epithelium off the matrix and process it for the isolation of proteins or RNA. RNA can be obtained from specific cell populations (e.g., epithelial cells, fibroblast regions in the matrix) using laser capture microdissection (LCM), followed by *in vitro* RNA amplification and microarray analysis or quantitative reverse transcription PCR (Kalabis et al., 2012). The goal of OTC is to create a more physiologically relevant environment that better represents the interactions between different cell types within the esophagus, which allows researchers to study how the tumor microenvironment influences cancer progression and to test potential drugs or therapies in a more realistic context (Liu and Wang, 2023). Creating accurate co-culture models requires careful selection of relevant cell types and optimizing culture conditions. Validating the findings from a co-culture system is crucial to ensure their relevance and accuracy in reflecting the *in vivo* situation.

#### 2.2.3 Sphere formation

Sphere formation assays gained popularity in the 2000s as an excellent tool to characterize stem cells from multiple tumors (Reynolds and Weiss, 1992; Weiswald et al., 2015). Spheroids mimic tumor-like conditions and enable the study of cell–cell interactions, drug penetration, and resistance. These assays have been used in cancer studies to look at cancer stem cells (CSCs) or tumor-initiating cells along with many possible CSC markers and to evaluate these cell populations' responsiveness to therapy (Whelan et al., 2018). Metformin has been shown to inhibit sphere formation in Aldehyde dehydrogenase (ALDH)1+ esophageal adenocarcinoma CSCs through the phosphatidylinositol 3-kinase/AKT and mammalian target of rapamycin pathways (Honjo et al., 2014). γ-secretase inhibitors impaired tumor initiation as well as sphere formation by EAC CSCs through inhibition of Notch signaling (Wang et al., 2014). Spheroids offer several advantages due to their simplicity, shorter culture time, and cost-effectiveness compared to organoids. 3D spheroid cultures are considered superior for representing tumors due to several key factors including: 1) Cell-Cell and Cell-Matrix Interactions: In 3D cultures, cells can interact with each other and the extracellular matrix in a manner that closely mimics the natural tumor environment (Białkowska et al., 2020). 2) Nutrient and Oxygen Gradients: 3D spheroids can develop gradients of nutrients and oxygen similar to those found in actual tumors. This includes hypoxic (low oxygen) regions, which are common in solid tumors and influence tumor growth and drug resistance (Khafaga et al., 2022). 3) Phenotypic Heterogeneity: 3D cultures allow for the formation of different cell layers, including proliferating outer layers and quiescent or necrotic cores. This heterogeneity is a hallmark of tumors and affects how they respond to treatments (Senrung et al., 2023). 4) Drug Response and Resistance: the complex environment of 3D cultures can better predict how tumors will respond to drugs, including the development of resistance. This makes 3D models more reliable for testing the efficacy and safety of new treatments (Zhang et al., 2023). 5) Mimicking the Tumor Microenvironment: 3D cultures can replicate the interactions between tumor cells and their surrounding microenvironment, including stromal cells and immune cells. This is essential for understanding tumor biology and developing effective therapies (Khafaga et al., 2022).

Overall, 3D spheroid cultures provide a more accurate and comprehensive model of tumor behavior, making them invaluable for cancer research and drug development. However, their simplicity also come with limitations: spheroids lack polarity and do not always fully represent the *in vivo* environment (Białkowska et al., 2020).

#### 2.2.4 3D organoids

Organoids are emerging as a prominent cell culture method across various biomedical research endeavors. Their diverse tissue origins, ability for long-term expansion, and physiological 3D structure render them a potent technology for numerous biological and clinical pursuits (Corrò et al., 2020). EAC Organoids are 3D structures that simulate the biology of the esophagus (Nakagawa et al., 2020). By mimicking epithelial architecture, interactions with the extracellular matrix (ECM), and tumor cell heterogeneity, EAC organoids provide a more accurate tumor model for studying disease progression and therapeutic resistance compared to traditional 2D culture systems (Li et al., 2018). Nowadays, it is common practice to employ organoids produced from various mice or human tumors to examine different cancer types (Corrò et al., 2020). The emerging 3D organoid technology, capable of growing matched normal and tumor patient-derived organoids (PDOs) enables comprehensive evaluation of drug toxicity and offers the potential to identify optimal doses that effectively eliminate tumor cells while minimizing harm to healthy tissue (Corrò et al., 2020). Another vital clinical use of PDOs is in screening for drug responders. Furthermore, PDOs have been employed for exploring drug combination approaches (Pauli et al., 2017) and assessing responses to chemotherapy and radiotherapy (Pasch et al., 2019). Liu et al. (2018) utilized CRISPR-Cas9 technology to generate Wnt-activated human BE organoid models, revealing that Wnt signaling activation leads to increased proliferation, replication capacity, and reduces apoptosis compared to wild-type organoids. Kunze et al. (2020) explored the Notch pathway’s impact on BE goblet cells, finding that Notch activation decreases goblet cell density and is closely linked to NF-κB activation, highlighting NF-κB-mediated inflammation as a key factor in tumorigenesis. In the study by Liu et al. (2018), various drugs, including 5-fluorouracil, epirubicin, and cisplatin, were tested on nine EAC PDOs. The findings indicated that the organoid model exhibited adverse clinical effects comparable to actual treatments, a result also noted by Derouet et al. (2020). The EAC PDO effectively mirrored the drug resistance seen in real tumors and showed different levels of resistance to various chemotherapeutic agents. While organoid technology serves as a notable intermediary between cell lines and *in vivo* models, there remain constraints within the current system. Despite their inherent heterogeneity, many PDOs are deficient in surrounding stromal cells within the culture, thus failing to fully replicate the tumor microenvironment (TME). This absence of TME within PDOs could potentially compromise their utility in predicting clinical outcomes accurately (Corrò et al., 2020). In an effort to overcome this limitation, a number of recent studies have tested the inclusion of immune cells in the organoid culture system (Corrò et al., 2020). Additionally, there are size variations in 3D models, which affects how repeatable the data is. Subsequently, non-uniform cell attachment and the absence of high-throughput techniques for tumor model formation are constraints associated with 3D models (Ju, 2018).

#### 2.2.5 Organ-on-a-chip

A recent example of organ-on-a-chip technology is the multichannel microfluidic perfusion culture system. This technique uses microfluidic devices made of glass, plastic, or man-made polymers. The system is made up of different sections that hold different kinds of cells, such as mesenchymal and endothelial cells, either with or without extracellular matrix (ECM). These platforms make it possible to study how cancer cells interact with the surrounding stroma while maintaining the structural integrity of living tissues (Trujillo-de Santiago et al., 2019). It has become possible to analyze the early phases of tumor growth and the formation of the tumor microvascular network by using microfluidic devices (Li et al., 2021). These small-scale devices effectively replicate the physiology and pathophysiology of specific human organs, closely mirroring conditions within the human body (Hassell et al., 2017). These models are considered superior to traditional 2D *in vitro* approaches given their complexity and capability of mimicking the structure and function of an organ (Joseph et al., 2022). In contrast to *in vivo* models, their affordability and potential for animal-free experimentation are expected to encourage widespread adoption, particularly in cancer research and the exploration of therapeutic avenues (Nahak et al., 2022). Recently, Shimshoni et al. (2023) developed an *in vitro* model using Organ Chip technology that mimics how the epithelium of Barrett’s esophagus (BE) responds to stroma-derived fibroblasts in a patient-specific manner. Although the current model is oversimplified, future enhancements could introduce additional microenvironmental complexity, including diverse immune cells, specific extracellular matrix molecules, and vascular endothelium. These additions would allow for a comprehensive study of the varied stromal contributions to BE and EAC pathobiology in the future.

## 3 Animal models of EAC

Animal models have enabled the simulation of human diseases and testing therapeutic approaches in ways that are not possible in human subjects. Unlike *in vitro* models, animal models can replicate the complexity of whole organisms, including interactions between different tissues, organs, and systems. Establishment of adequate *in vivo* models is necessary for understanding the mechanisms underlying esophageal adenocarcinoma (EAC) development and progression. In this section, we summarize the EAC animal models that have been developed so far.

### 3.1 Rat models

The surgical reflux rat model was developed by Levrat et al. (1962) to study esophageal adenocarcinoma. In this model, an esophagoduodenal anastomosis (EGDA) is performed to expose the esophageal epithelium to duodenal bile salts and evaluate their contribution to tumor progression. Rats are considered suitable for this model due to their larger size compared to mice and similar esophageal adenocarcinoma pathophysiology as compared to humans (Nair and Reddy, 2016). The EGDA rat model became the standard for surgical models (Matsui et al., 2017; Tang et al., 2021). Major limitations of surgical models include unpredictable rate of model formation, time-consuming and traumatic procedure, and high mortality rate (Tang et al., 2021). In addition, there are significant concerns regarding the translatability of these studies to human disease for several reasons including histological differences between mouse and human esophagi (Horn et al., 2013), unpredictable reproducibility with unexpected development of mixed tumor types (both adeno and squamous tumors) (Hashimoto, 2012), and absence of deep invasion and metastasis questioning whether the induced malignancies differ in their aggressiveness from human disease (Kapoor et al., 2015). Hence, a better model is needed to address these issues.

### 3.2 Mouse models

**FIGURE 2:**
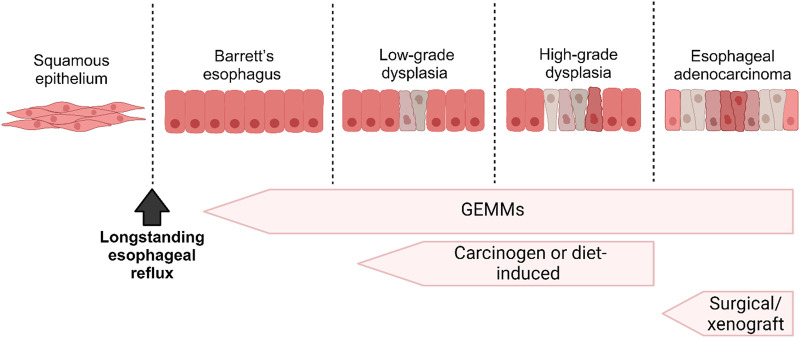
A schematic diagram illustrating the progression from Barrett’s Esophagus (BE) to Esophageal Adenocarcinoma (EAC) and correlating it with the various *in vivo* models employed to investigate each stage.

Many attempts have been made to establish mouse models for EAC research, starting with surgical approaches to genetically engineered models (Table 1; Figure 2). We have divided the mouse models below, based on the approach used.

**TABLE 1:** Mouse models of EAC.

Type of model		Mechanism	Pros	Cons	Ref.
Surgical models		Esophagoduodenal anastomosis with total gastrectomy to expose the esophagus to duodenal content reflux	• Mimics human reflux • Cost-Effective compared to other mouse models	• Difficulty in performing surgery • Time-consuming • High mortality rate due to intolerance of surgical stress • Unpredictable reproducibility • Unexpected development of mixed tumor types	Pham et al. (2014), Ellis et al. (2001), Aikou et al. (2013), Davelaar et al. (2015)
Xenograft models	Subcutaneous	Injection of cancer cells under the skin of immunodeficient mice	• Allow direct assessment of tumor growth • Preserve reproducibility • Relatively inexpensive • Technically simple	• Large number of cells needed for injection • Lack of tumor heterogeneity • Use of immunodeficient mice does not allow the study of tumor cell-host immune response interaction • No metastasis formation	Ruggeri et al. (2014), Mahmoudian et al. (2021)
	Orthotopic	Implantation of esophageal cancer cells in the upper or lower end area of the esophagus of immunodeficient mice	• More closely resembles human esophageal cancer progression • Frequently forms distant metastases	• Difficulty in performing the implantation due to the anatomical size and location of the mouse’s esophagus • Lack of immune response • Requires specialized diagnostic techniques for monitoring tumor growth • Time and labor-intensive • High-cost procedure	Bibby, 2004; Gros et al. (2010a), Tétreault (2015)
	Patient-derived xenograft (PDX)	Engraftment of patient’s tumor biopsies into immunodeficient mice	• Retain the original tumor’s architecture and stromal components • Maintain tumor heterogeneity • Original tissue can be serially propagated *in vivo*	• Inability to study tumor-host interaction due to the use of immunodeficient mice • Long latency phase for growing the tumor • Dependency of engraftment rate on numerous factors	Cho et al. (2016), Damhofer et al. (2015), Dodbiba et al. (2015), Lan et al., 2021; Cho (2020), Barra et al. (2017), Nakauchi et al. (2023), Cellini et al. (2014), Veeranki et al. (2019a), Lee et al. (2018)
Carcinogen or diet-induced models		Treatment of mice with carcinogens including NMBA, DCA and MNU or diet deficient in specific nutrients such as zinc	• Mimic environmental exposures associated with human esophageal cancer • Can be combined with GEMM to facilitate EAC development	• Treatment outcome is influenced by mouse’s genetic makeup • No metastasis formation • Requires the handling of chemicals • Cancer phenotypes are heterogeneous	Kemp (2015), McQuaid et al. (2011), Guy et al. (2007)
Genetically engineered mouse models (GEMMs)		Loss or gain of function of specific genes	• Spontaneous development of tumors in their native microenvironment • Mice are immunocompetent	• Heterogeneity in the frequency and tumor growth • Long latency • Limited options to express or inhibit genes in esophagus • Slow cancer progression rate in mice • Development of cancer is influenced by the genetic background of the mice • Rare incidence of invasion and metastasis	Mahmoudian et al. (2021), Quante et al. (2012), Chavan (2013)

#### 3.2.1 Surgical models

The development of models to mimic reflux conditions using the EGDA approach in mouse models has been challenging, mainly due to difficulty in performing surgery on mice due to their small size and the high mortality rate associated with the mice’s intolerance to surgical stress. In addition, there is a much lower incidence of Barrett’s esophagus (BE) or EAC as compared to rat reflux models even with the addition of exogenous carcinogens (Kapoor et al., 2015). Nonetheless, there are a number of EAC studies that utilized this mouse model (Pham et al., 2014; Ellis et al., 2001; Aikou et al., 2013; Davelaar et al., 2015; Caspa Gokulan et al., 2023). These reported studies showed 6%–50% incidence of EAC with an overall mortality ranging from 4% to 30%. The onset of BE or EAC in these studies required relatively long time ranging from 20 to 40 weeks following surgery (Pham et al., 2014; Ellis et al., 2001; Aikou et al., 2013). Davelaar et al. (2015) established a novel suture-less method to create the EGDA model by implanting neodymium micromagnets in both esophagus and jejunum which then oppose fistulation within days by pressure necrosis. This approach was associated with lower morbidity and mortality and accelerated the onset of BE lesions to 9 weeks in 50% of mice.

#### 3.2.2 Xenograft models

These models are generated by transplanting cultured esophageal cancer cells or patient tumor tissues into immunodeficient mice. These models are divided into the following three groups based on the site of transplantation and type of sample: subcutaneous or ectopic xenograft, and orthotopic xenograft.

##### 3.2.2.1 Subcutaneous or ectopic xenograft

The subcutaneous xenograft model is generated by injecting human esophageal cancer cell lines under the skin of immunodeficient mice (Ruggeri et al., 2014). This is an old method that has been extensively used to study the biology and mechanism of esophageal cancer tumorigenesis *in vivo* (Mahmoudian et al., 2021). Subcutaneous xenograft models allow direct assessment of tumor growth, can preserve reproducibility and are relatively inexpensive and technically simple to develop (Ruggeri et al., 2014; Mahmoudian et al., 2021). Nonetheless, this approach has some limitations that include the large number of cells needed for each injection, the lack of tumor heterogeneity, and the use of immunodeficient mice, which makes it inappropriate to study tumor cell–host immune response interactions and metastasis (Ruggeri et al., 2014; Mahmoudian et al., 2021).

##### 3.2.2.2 Orthotopic xenograft model

The difference between subcutaneous and orthotopic xenograft models is the site of injection. In the orthotopic model, esophageal cancer cells or fragments are implanted in the upper or lower end area of the esophagus of immunodeficient mice (Song et al., 2014). This model is considered a better option for studying esophageal cancer than the subcutaneous model because it more closely resembles human esophageal cancer progression given the fact that tumor grow in a native tumor microenvironment and it frequently forms distant metastases (Bibby, 2004). Gros et al. (2010a) used OE19 and PT1590 cell lines to successfully generate a highly metastatic orthotopic EAC mouse model that showed metastatic spread to the liver, lungs, and lymph nodes. Among the limitations of orthotopic mouse models is the requirement of specialized diagnostic techniques such as bioluminescent imaging for monitoring tumor growth (Tétreault, 2015). Transfection with luciferase reporter gene combined with bioluminescent imaging has been commonly used to overcome this limitation and monitor tumor growth noninvasively (Kuchimaru et al., 2016). In addition, Gros et al. (2010a) used high-resolution imaging with green fluorescent protein and magnetic resonance imaging to noninvasively monitor tumor growth and evaluate therapeutic responses to treatment. Additional weaknesses of this model include technical difficulties in performing the implantation due to the anatomical size and location of the mouse’s esophagus, lack of immune response, time and labor-intensive, and high-cost procedure (Tétreault, 2015).

##### 3.2.2.3 Patient-derived xenografts (PDXs)

PDXs are a good example of valuable xenograft models used in cancer research. PDX models are developed by implanting a patient’s tumor biopsy into an immunodeficient mouse subcutaneously or orthotopically (Cho et al., 2016). Among the documented studies establishing preclinical models of esophageal cancer, PDX models derived from esophageal squamous cell carcinoma (ESCC) patients were more commonly observed compared to those derived from EAC patients. Damhofer et al. (2015) successfully established 18 EAC PDX models and validated their clinicopathological features. Dodbiba et al. (2015) achieved a success rate of 38% in establishing 21 PDX models from esophageal/gastroesophageal junction cancers, and among 7 xenografts subjected to drug testing, only 2 exhibited chemosensitivity correlating with clinical responses. Nonetheless, PDX models, with reported engraftment rates ranging from 13.3% to 55.5% (Lan et al., 2021), provide a valuable platform for studying EAC. Dodbiba et al. (2015) revealed that PDX models exhibit aggressive characteristics, particularly in poorly differentiated tumors and older patients, leading to higher engraftment rates. Additionally, they found consistent gene and protein expression profiles across various passages of the PDX models between patient samples and corresponding xenografts, indicating the preservation of tumor characteristics in the PDX models (Dodbiba et al., 2015). PDXs are crucial in personalized medicine, yet their effectiveness can be limited when implanted at subcutaneous sites due to differences in anatomy and tumor microenvironment compared to gastrointestinal tracts (Cho, 2020). Gastroesophageal junction (GEJ) cancers, known for their aggressiveness (Barra et al., 2017; Nakauchi et al., 2023), possess unique characteristics that make subcutaneous PDX models inadequate for studying certain aspects of tumor growth and treatment response (Cellini et al., 2014). To address this, Veeranki et al. (Veeranki et al., 2019a) developed a GEJ patient-derived orthotopic xenograft (PDOX) model, implanting cancer cells directly at the mouse GEJ to closely mimic human tumors.

When compared to other xenograft models, PDXs are more reliable for the study of esophageal cancer because they retain the original tumor’s architecture and stromal components and maintain tumor heterogeneity (Lee et al., 2018). The key disadvantages of PDX models include the inability to study tumor-host interaction due to the use of immunodeficient mice, the long latency phase for growing the tumor, the dependency of engraftment rate on numerous factors such as mouse strain, tumor and patient features, region of tumor implantation, and tumor type (Ruggeri et al., 2014; Cho et al., 2016).

#### 3.2.3 Carcinogen or diet-induced models

Treatment with carcinogens such as 4-Nitroquinoline 1-oxide (4-NQO), N-nitrosomethylbenzylamine (NMBA), Deoxycholate (DCA), and N-Methyl-Nnitrosourea (MNU) or diet manipulation have been used to induce esophageal carcinogenesis in mouse models (Nair and Reddy, 2016). Among the limitations of these models are the inability to study metastasis, the response to treatment is affected by the mouse’s genetic makeup, the susceptibility to carcinogens and resultant tumor incidence and multiplicity is influenced by several factors including the dose and schedule of the carcinogen, the age and the strain of the mouse used (Kemp, 2015). The unconjugated bile acid, DCA, MNU, as well as zinc-deficient diet combined with NMBA or DCA have all been used to induce esophageal carcinogenesis. BE lesions were observed at low frequency and progression to EAC was rarely reported in these models (McQuaid et al., 2011; Guy et al., 2007). To facilitate the development of EAC, the combination of carcinogens with genetically engineered mouse models has been applied as detailed in the following section.

#### 3.2.4 Genetically engineered mouse models (GEMMs)

These models are generated by genomic manipulation to investigate mechanisms of tumor formation and identify potential therapeutic agents (Le Magnen et al., 2016). They include transgenic mouse models, gene knockin and knockout models, and conditional/inducible models. The development of EAC is a multistep process that involves replacement of squamous epithelium with glandular one along with activation or suppression of specific genes. Therefore, the generation of GEMMs, while challenging, is an important tool for the identification of the molecular mechanisms involved in this disease (Yue et al., 2017). The development of GEMMs of EAC has also been extremely challenging due to the histological differences between the esophagus of mice and humans and the time course of EAC development in humans, which explains the fact that the majority of EAC mouse models are xenograft models and very few GEMMs exist (Tetreault et al., 2012; Lehman and Stairs, 2015). Here, we review the GEMMs of EAC that have been developed so far with their advantages and limitations.

GEMMs of BE and EAC have been developed using the ED-L2 and K14 promoters to target specific genes implicated in the progression of esophageal cancer including P53, P14, P16, CDX2, IL1β, and ErbB2. Hao et al. (2009) attempted to model EAC using P53, P16, and P14 knockout mice along with gastroesophageal reflux and iron treatment. The mice developed metaplasia and squamous cell carcinoma; however, none of the mice developed EAC. Xie et al. (1999) showed that the conditional overexpression of ErbB2 in mice can induce severe hyperplasia and irregularity in the basal layer of the esophagus. The P27-deficient mouse model in combination with N-methyl-N-benzylnitrosamine (MBN) administration and gastroduodenal-esophageal reflux developed by Lechpammer et al. (2005) showed BE development, but no progression to EAC. Mckeon et al. (Wang et al., 2011) developed a P63-deficient neonatal mouse model characterized by the development of BE like columnar epithelium. However, deletion of P63 in germ line led to early lethality of mice and limited the potential use of this model to study the pathogenesis of BE and EAC. Jiang et al. (2017) showed that the activation of CDX2 in the transitional basal cells at the squamous-columnar junction was sufficient to generate BE metaplasia phenotype providing evidence on the cell-of-origin for BE. Quante et al. (2012) developed an innovative approach to generate a genetic mouse model of inflammation-dependent BE and EAC. They used the ED-L2 promoter to drive the transgenic expression of the proinflammatory cytokine interleukin (IL)-1β to the esophageal and squamous forestomach mucosa of mice, resulting in inflammation by 6 months of age, severe metaplasia by 12–15 months of age and high-grade dysplasia or EAC by 20–22 months of age. Treatment of IL-1β-overexpressing mice with bile acids alone (0.2% deoxycholic acid in drinking water) or in combination with N-Methyl-N-nitrosourea (MNU) markedly accelerated the onset of BE and EAC. Thus far, this model has improved the understanding of potential origin and pathogenesis of BE and EAC.

GEMMs have numerous advantages over other models, including defined genetic background, spontaneous development and normal growth rate of tumors in their native microenvironment. In addition, they maintain an active immune system where conditional knockout or activation of genes allows studies of spatial and temporal control of gene activity (Mahmoudian et al., 2021; Chavan, 2013). However, these models have limitations among which are the relatively mild phenotypes due to slow cancer progression rate in mice, heterogeneity in the frequency and tumor growth, long latency, limited options to express or inhibit genes, and the rare incidence of invasion and metastasis (Mahmoudian et al., 2021; Singh et al., 2012).

## 4 Discussion

Esophageal adenocarcinoma remains one of the most understudied malignancies, primarily due to the limited availability of model systems that can adequately represent its pathogenesis and enable effective drug testing (Katt et al., 2016). Despite these challenges, the ongoing efforts have led to the development of some models that aid in studying EAC pathogenesis, evaluating potential therapeutic targets, and testing novel treatments. In this review, we discuss different *in vitro* and *in vivo* models that are available for studying EAC and summarize their advantages and disadvantages.

*In vitro* models continue to play a crucial role in advancing our understanding of EAC biology, drug responses, and potential therapeutic targets. There is a wide range of *in vitro* models, each with its unique strengths and weaknesses (Katt et al., 2016). Given the inherent differences in complexity and functionality, the selection of a model often relies on the specific application. Recent advancements in tumor cell biology, 3D cell culture, tissue engineering, biomaterials, microfabrication, and microfluidics have facilitated the rapid development of *in vitro* tumor models (Abuwatfa et al., 2024). These novel models exhibit increased complexity compared to traditional ones by incorporating multiple cell types (coculture), extracellular matrix materials (ECM), and the spatial and temporal introduction of soluble factors (Jubelin et al., 2022). Additionally, innovative approaches now include the incorporation of perfusable microvessels to simulate tumor vasculature, which plays a crucial role in cancer progression and drug transport (Jubelin et al., 2022). Esophageal 3D culture systems including OTC and organoids have provided substantial molecular and mechanistic insights into EAC development and progression. Researchers have studied BE-derived cell lines in OTC, where all-trans-retinoic acid (ATRA) was observed to impact the transition from squamous-like multilayered epithelial cells to columnar epithelial cells (Kosoff et al., 2012). Additionally, inflammatory molecules like interleukin-1β and COX-2 have been implicated in BE development, and COX-2 overexpression was associated with the formation of intestinal mucin-filled epithelia (Kong et al., 2011). OTC has been used to characterize and study the invasiveness of several EAC cell lines (OE19, OE33, FLO-1, and MDF-1) (Kalabis et al., 2012; Underwood et al., 2015; Kalabis et al., 2008; Lu et al., 2023; Chen et al., 2023). Underwood et al. (Underwood et al., 2015) showed that cancer-associated fibroblasts promote invasion via fibroblast-derived periostin in EAC. Lu et al. (2023) and Chen et al. (2023) showed that exposure of OTC to acidic bile salts that mimic reflux conditions in patients induced E-cadherin cleavage and upregulated MMP14, APE1 and DLL1 thus promoting epithelial-mesenchymal transition and stem-like properties in EAC. OTC was also used as a testing platform for molecularly targeted therapeutics including EGFR, mutant p53, and PIK3CA (Whelan et al., 2018). Significant advancements have been made in the establishment and characterization of patient derived EAC organoids (Li et al., 2018; Karakasheva et al., 2020). These organoids recapitulated the histology and heterogeneity of the original tumors providing a model for clonality studies and precision therapeutics (Li et al., 2018; Karakasheva et al., 2020). Addition of immune cells and potentially other nonepithelial components in OTC and organoid cultures may open new avenues of research and preclinical drug testing. The emergence of 3D tumor culture systems is bridging the gap between *in vitro* and *in vivo* methods for drug screening, as these 3D models continue to improve as reliable indicators of *in vivo* drug efficacy (Abuwatfa et al., 2024).

Appropriate *in vivo* models are required to adequately mimic the molecular, functional, and phenotypic characteristics of human tumors. The ideal animal model for EAC should take the following criteria into consideration: genetic relevance to human, conserved histological architecture, naturally occurring pathophysiological GERD, molecular validation for similarity in pathogenic progression and practical feasibility (Attwood et al., 2008). Although the perfect EAC mouse model does not exist, a few models have emerged over the years that provided valuable insight into esophageal tumor biology despite their limitations and challenges (Kapoor et al., 2015). The highly aggressive orthotopic EAC model developed by Gros et al. (2010a) was used as a preclinical tool to evaluate the chemotherapeutic effects of targeted therapies against HER-2 (Gros et al., 2010b) (a member of the EGFR family) and the C-X-C motif chemokine receptor type 4 (CXCR4) antagonist CTCE-9908 (Drenckhan et al., 2013) in esophageal cancer. The ED-L2/IL-1β mouse model combined with unconjugated bile acids treatment is one of the most used *in vivo* models in EAC research. This model has improved understanding of the potential cellular origin of BE in which researchers showed that BE can arise from gastric progenitors that are positive for Lgr5, and that IL-6 deficiency inhibited the development of BE and EAC suggesting a role for inflammation in inducing esophageal metaplasia and the progression of esophageal carcinogenesis (Quante et al., 2012). Using this model, researchers have demonstrated a role for bile acids, APE1, and NOTCH signaling in the pathogenesis of EAC (Lu et al., 2023; Chen et al., 2023; Ballout et al., 2022). EAC-PDX models became useful tools in translational cancer research. Veeranki et al. (2019b) showed that inhibition of CDK9 using BAY1143572 could sensitize EAC PDX models to radiation. Teichman et al. (2018) reported that hedgehog ligands are upregulated in the tumor epithelium of EAC PDX models and that inhibiting hedgehog signaling mediates radiation sensitivity in these models. Recent studies showed that targeting APE1 using the redox inhibitor E3330 inhibits EAC PDX tumor growth and reduces EMT characteristics (Lu et al., 2023). In addition, combining SMAD3 inhibition with oxaliplatin treatment in EAC PDX models suppressed tumor growth by enhancing DNA damage (Ballout et al., 2024). However, several challenges persist in the establishment and utilization of EAC-PDX models including low engraftment rates (Miao et al., 2020), the lack of functional immune system (Miao et al., 2020), the replacement of human stromal cells by mouse stroma in the initial stage of PDX establishment (Damhofer et al., 2015), and the subcutaneous engraftment commonly used by researchers that inaccurately reflects tumor progression compared to orthotopic methods (Lee et al., 2018). While current EAC-PDX models have limitations, the emergence of novel immunodeficient animals such as humanized animal models could enhance their utility in preclinical studies.

When comparing 3D models and mouse models in research, there are several factors to consider, including time and cost. Creating 3D models, such as organoids or tissue cultures, can be relatively quick, often taking days to weeks to develop. These models can be rapidly reproduced once the initial setup is complete (Urzì et al., 2023). On the other hand, developing genetically engineered mouse models can take several months due to breeding cycles and the time required for genetic modifications (Gurumurthy and Lloyd, 2019). Additionally, longitudinal studies in mice can extend over months or even years. When it comes to cost, the expense of 3D models can vary widely depending on the complexity and type of model; however, they are generally more cost-effective than mouse models, especially when considering the reduced need for housing and long-term care (Urzì et al., 2023). Other factors to consider include ethical concerns and biological relevance. 3D models often present fewer ethical concerns compared to animal models, as they do not involve live animals. In terms of biological relevance, mouse models have an advantage over 3D models because they provide whole-organism insights, which are crucial for understanding complex biological systems and disease mechanisms (Gurumurthy and Lloyd, 2019).

Modeling EAC *in vivo* is challenging due to the fundamental histological differences between human and mouse esophagus, the lack of model systems reflecting the primary stages of the disease, low rate of mice survival to maturity (less than 20%), and the poor recapitulation of human tumors through mice tumor models (Nair and Reddy, 2016; Tratar et al., 2018). Improvisation of EAC reproducibility in mice models is instrumental for future molecular studies on EAC pathogenesis and metastasis, two areas that are currently under investigated. Given that genomic analyses of EAC have identified a mutational signature for this type of tumor (Tétreault, 2015), the generation of future GEMMs should focus on assessing the effects of the mutated genes' expression in EAC as well as on having a faster progression to the development of invasive cancer phenotypes. The lack of syngeneic mouse models is another major drawback in modeling EAC *in vivo*. These models play a crucial role in understanding tumor microenvironments and are particularly valuable for studying how cancer therapies perform in the presence of a functional immune system (Tétreault, 2015). To maximize the potential of current mouse models of EAC, the combined use of multiple models including human samples, more than one type of mouse model, and the near-physiological esophageal tissue organoid model is highly recommended. Moving forward, structural and functional differences between human and rodent need cautious consideration and the use of higher animal models should be endeavored. Because pigs are evolutionarily close to humans, they have been extensively utilized in biomedical research (Abdulnour-Nakhoul et al., 2007; Groenen et al., 2012; Hai et al., 2014). No successful swine BE or EAC model has been reported so far; however, this approach may bridge the gap between primates and rodents in terms of translatability of research findings and suitability for lab studies.

Incorporating immune cells into organoid cultures and developing organ-on-a-chip models are exciting advancements in the study of esophageal adenocarcinoma. Integrating immune cells into organoid models allows researchers to create more physiologically relevant systems that better represent human disease. This can be achieved through methods like immune cell injection, co-culture, and tissue expansion with existing immune cells (Bogoslowski et al., 2023). By including immune cells, researchers can gain valuable insights into how organs function under stress or disease, leading to a deeper understanding of disease mechanisms and potential therapeutic interventions. Moreover, organ-on-a chip models simulate the structure and function of human organs and offer a high-fidelity platform for studying diseases like esophageal adenocarcinoma. Researchers at the Wyss Institute for Biologically Inspired Engineering have developed an esophagus-on-a-chip model that can recapitulate the responses of esophageal epithelium to stroma-derived fibroblasts in a patient-specific manner (Shimshoni et al., 2023). This allows for more accurate disease modeling and personalized therapy development.

In summary, a comprehensive understanding of EAC requires a multifaceted approach that integrates *in vitro* and *in vivo* models. These models serve as valuable tools for advancing our knowledge of EAC and developing effective therapeutic strategies.
